# Relationship between Soybean Protein Isolate and Textural Properties of Texturized Vegetable Protein

**DOI:** 10.3390/molecules28227465

**Published:** 2023-11-07

**Authors:** Lin Li, Yatao Huang, Yanfang Liu, Yangyang Xiong, Xinrui Wang, Litao Tong, Fengzhong Wang, Bei Fan, Xiaojia Bai

**Affiliations:** 1 State Key Laboratory of Food Nutrition and Safety, Tianjin University of Science and Technology, Tianjin 300457, China; lilin13259354082@163.com; 2 Western Agricultural Research Center, Chinese Academy of Agricultural Sciences, Changji 831100, China; huangyatao@caas.cn (Y.H.); fanbei@caas.cn (B.F.); 3 Key Laboratory of Agro-Products Quality and Safety Control in Storage and Transport Process, Ministry of Agriculture and Rural Affairs, Institute of Food Science and Technology, Chinese Academy of Agricultural Sciences, Beijing 100193, China; liuyanfang054@163.com (Y.L.); 82101215459@caas.cn (Y.X.); wangxinrui202202@163.com (X.W.); tonglitao@caas.cn (L.T.)

**Keywords:** soybean protein isolate, functional property, extrusion, textural properties

## Abstract

To identify the ideal soybean protein isolate for texturized vegetable protein processing, the effect of different soybean protein isolates on texturized vegetable protein composition was studied. Three different types of soybean protein isolates were selected and analyzed for functional properties (water holding capacity (WHC), emulsifying properties, foaming properties), amino acid content, and protein secondary structure. Then, using the same formulation, the soybean protein isolates were extruded to produce texturized vegetable protein, and its textural properties, degree of texturization, microstructure, free sulfhydryl (free SH), and disulfide (S-S) content were determined. Lastly, a correlation analysis was performed to examine the connection between soybean protein isolates and texturized vegetable proteins. After correlation analysis, the soybean protein isolate functional properties that affect the textural properties of the texturized vegetable protein were as follows: the emulsifying property affected the hardness, adhesiveness, springiness, gumminess, and chewiness of the texturized vegetable proteins; and the foaming property affected the gumminess, chewiness, and the degree of texturization of the texturized vegetable proteins. In addition, 16 amino acids including threonine (Thr), methionine (Met), and arginine (Arg) affect texturized vegetable proteins, mainly with respect to adhesiveness, springiness, and free SH. The effects of secondary structure (α-helix, random coil) on texturized vegetable proteins were degree of texturization, resilience, and cohesion, respectively. Therefore, choosing the soybean protein isolate with better emulsifying and foaming properties provides a more suitable approach for processing texturized vegetable protein.

## 1. Introduction

Texturized vegetable proteins are healthy, sustainable [1], and can meet the protein needs of a growing population [2,3]. Nutritionally, it is high in protein, low in calories, low in fat, and zero in cholesterol [4]. As a result, texturized vegetable proteins are gaining consumer attention and providing better options for vegetarians [5]. Texturized vegetable protein is formed by extrusion technology similar to animal meat, with abundant fiber and texture similar to animal meat [6], which has been more studied in recent years [7]. Some researchers have found that the main influences on textural properties of texturized vegetable protein include raw materials [7], additives such as polysaccharides [8,9,10] and enzymes [11], and extrusion conditions [12,13], among which raw materials are the key influencing factors [14].

At present, the main raw materials used in the production of texturized vegetable proteins are soybean protein [15], peanut protein [16], pea protein [17], and wheat gluten [18]. Previous studies have found that different raw materials have different effects on texturized vegetable protein. Using soybean protein as the main ingredient, a series of physical and chemical changes are produced through the extrusion process, resulting in the formation of texturized vegetable protein [19]. Texturized vegetable proteins possess unique layered and fibrous structures, exhibiting a significant level of fibrousness and an orderly structural orientation, which affects the digestibility and absorbability of the protein and its functional properties [20,21]. Due to its exceptional gelling properties, superior nutritional value, and cost-effectiveness, soybean protein is widely acknowledged as an ideal option for the development of texturized vegetable protein meat substitutes [14]. A few studies have used defatted peanut protein for texturizing under low-moisture conditions [22,23]. The reduced capacity of peanut proteins to form a rich fiber structure during extrusion is primarily due to their inferior gel-forming ability compared to soybean proteins [20,22]. The researchers discovered that the primary forces responsible for maintaining the structure of peanut proteins are non-covalent bonds, such as hydrophobic interactions and hydrogen bonds, with disulfide bonds playing a secondary role [24]. Consequently, in contrast to soybean protein, peanut protein exhibits inadequate gelling capacity and lacks the required tensile strength and fibrous structure. As a result, it possesses a soft fibrous texture [16,25]. Pea proteins with a moisture content of 55% and a distinct fibrous structure can be extruded to form texturized vegetable protein [26]. However, the susceptibility of pea proteins to damage during extreme processing, such as high temperatures and rapid heating rates during extrusion, undermines their gelling properties [27,28]. Therefore, when pea protein is the main raw material, attention needs to be paid to the extrusion parameter (barrel temperature) settings in order to obtain properly texturized vegetable protein [29]. Compared to soybean protein, wheat protein lacks lysine and cannot meet the human demand for amino acids [30]. Due to its viscoelastic properties, wheat gluten can be individually formed into a continuous network structure by high moisture extrusion [31]. In terms of network structure formation, the processing properties of wheat gluten surpass those of other plant proteins due to its exceptional gelling properties [32]. To improve the fiber structure, wheat gluten is usually blended with other vegetable proteins such as soybeans, peanuts, and peas [33,34]. Therefore, in this paper, we chose to focus on different soybean protein isolates to investigate the effect of different soybean protein isolates on texturized vegetable proteins.

Texturized vegetable proteins are affected differently by different soybean proteins. The different compositions of soybean proteins (7 s and 11 s globulins) determine their various nutritional and functional properties such as moisture, surface, and protein mechanisms [35,36]. It has been shown that various soybean protein fractions can alter the textural properties, WHC, and rehydration properties of texturized vegetable protein [37]. Adding 7 s improves protein hydration properties, as well as foaming and emulsifying properties [38]. Adding 11s increases the structural strength of proteins and improves properties such as gelling [39]. The best textural properties of texturized vegetable protein were obtained at an 11 s/7 s ratio of 1.5 [40]. In addition, the interactions between proteins are an important determinant of solubility, gelling, adhesion, and other properties. There are significant contributions from hydrogen bonds, disulfide bonds, and hydrophobic interactions in the processing of texturized vegetable protein, which are the primary contributors influencing its textural properties [15,41,42]. Studies have found that the preservation of texturized vegetable proteins, regardless of whether soybean protein is processed at low or high moisture levels, relies on a combination of hydrophobic interactions, hydrogen bonds, disulfide bonds, and their synergistic interactions [15]. In this process, non-covalent bonds play a more significant role than covalent bonds. Similarly, the structure of proteins has an effect on the properties of texturized vegetable proteins. Research has shown that the presence of L-cysteine can alter the interconnection of disulfide bonds between protein molecules, resulting in noticeable changes in the texture and structure of textured vegetable proteins. The addition of L-cysteine may have an effect on the binding interactions within protein molecules, thus affecting their cross-linking effect [43]. Up to now, however, it has not been clear which properties and which structures of the raw material influence the texture of the texturized vegetable protein.

Therefore, to enhance the structural characteristics of texturized vegetable proteins, we established a specialized raw material index system. We first studied three different soybean protein isolates for functional properties (WHC, emulsifying properties, and foaming properties) and structure (amino acid composition and secondary structure). Then, we prepared three texturized vegetable proteins through low-moisture extrusion and analyzed their textural properties, degree of texturization, protein free SH and S-S content, and microstructure. Finally, we analyzed the correlation between soybean protein isolates and texturized vegetable proteins and drew relevant conclusions, which provide support for dedicated raw materials for texturized vegetable proteins.

## 2. Results and Discussion

### 2.1. Soybean Protein Isolate

#### 2.1.1. Soybean Protein Isolate’s Functional Properties

Functional properties of soybean protein isolate are very important for their use in a variety of markets, and their functional properties include WHC, emulsification properties (Emulsification Activity Index (EAI) and Emulsification Stability Index (ESI)), and foaming (Foam Capacity (FC) and Foam Stability (FS)). Studying the effect of soybean protein isolate on texturized vegetable proteins requires a thorough examination of its functional properties. Results are shown in Table 1. The outcomes regarding the functional characteristics of three soybean protein isolates were as follows: the average value of WHC was 6.51%, the range of variation was 6.33~6.83%, and the coefficient of variation was 3.5%, with a significant difference among the three. The mean value of EAI was 22.36 m^2^/g, the variation was 19.62~25.28 m^2^/g, the coefficient of variation was 1.94%, the largest EAI was 950 E, the smallest EAI was 970 W, and the difference between the three was highly significant. The average value of ESI was 18.58 min, the variation was 16.30~20.35 min, the coefficient of variation was 8.47%, and there was no significant difference among the three. The mean value of FC was 1.47%, the variation was 1.34~1.68%, the coefficient of variation was 2.1%, and the largest FC was 971 W. The mean value of FS was 0.31%, the variation was 0.28~0.35%, the coefficient of variation was 10.12%, and there was no significant difference among the three.

The WHC of soybean protein isolates were higher than those of pea protein isolates previously studied [26]. The WHC of soybean protein isolates was higher than that of pea protein isolates, indicating that soybean protein isolates are more soluble than pea protein isolates. Proteins with higher solubility have good dispersibility and can form a good dispersion system, which also improves the WHC of proteins. Previous studies indicated that pea protein isolate had an EAI of 18.32 m^2^/g and an ESI of 25.98 min, a FC of 31.7%, and a FS of 22.5% [17]. While the EAI of pea protein isolate was lower than that of soybean protein isolate, the ESI of pea protein isolate was higher than that of soybean protein isolate when compared to soybean protein isolate. The emulsification properties of different plant proteins were different, mainly because 7 s globulin has a higher content of hydrophobic amino acids and a large number of hydrophobic zones on the surface, so it can be adsorbed on the surface of the oil droplet faster and form a highly elastic and ordered protein film on the surface of the oil droplet [44]. The foaming properties of proteins include FC and FS. The foaming property of a protein is the ability of the protein to be able to foam, while FS is the ability of the foam to remain stable [45]. Compared to previous studies [17], the foaming properties of soybean protein isolates were much lower than those of pea protein isolates. This may be related to the structure of soybean protein isolates and the interaction of their protein complexes at the water-air interface [46].

#### 2.1.2. Amino Acid Content of Soybean Protein Isolate

To illustrate the differences in protein composition of different soybean protein isolates, the contents of 16 amino acids were determined for each of the three soybean protein isolates. The composition of amino acids (g/100 g) for the three soybean protein isolates is presented in Table 2. The results showed that the soybean protein isolates had a well-balanced amino acid composition. The three soybean protein isolates had an average amino acid content of 78.4 g/100 g, with a range of 75.9~79.6 g/100 g. The coefficient of variation was 6.4%, indicating a moderate level of variability. There was no significant difference in the highest amino acid content, which was 970 W, among the three isolates. The three soybean protein isolates had high levels of Asp, Glu, Arg, Leu, and Lys, comprising approximately 55% of the total amino acids. However, the content of Met was relatively low, at less than 2%. The essential amino acids made up 34% of the total amino acid content in all three samples, with no significant difference observed. Comparing the amino acid content of soybean protein isolate with that of mung bean protein isolate [47], the individual amino acid content of soybean protein isolate was higher than that of mung bean protein isolate. Compared to rice protein isolate, rice protein had 40.3 mg/g (4.0 g/100 g) of Val, 35.7 mg/g (3.6 g/100 g) of Tyr, and 41.6 mg/g (4.2 g/100 g) of Alanine [13] than the amino acids in soybean protein isolate. In conclusion, whether compared with mung bean protein isolate or rice protein isolate, the amino acid content of soybean protein isolate is still more comprehensive, as a good choice for processing raw materials.

#### 2.1.3. Secondary Structure of Proteins

**Figure 1:**
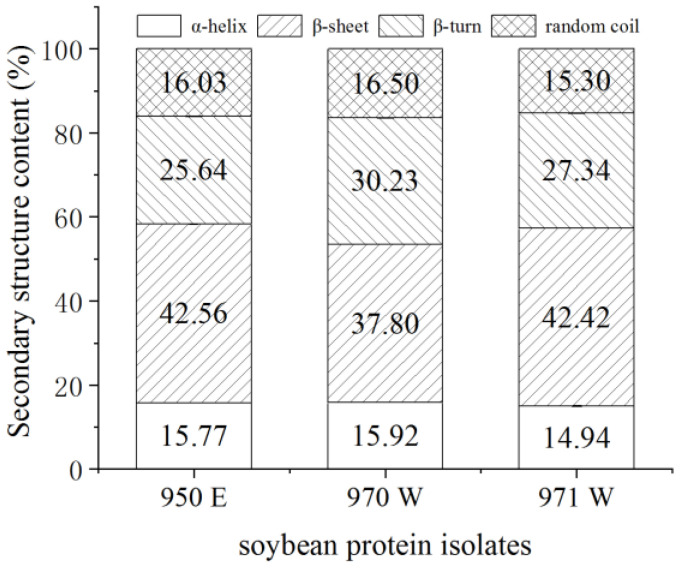
Content of secondary structure components of soybean protein isolates.

The changes in the secondary structure of soybean protein isolates in infrared spectroscopy primarily occur through modifications in the sub-peak content within the 1600–1700 cm^−1^ range of the amide I band [8]. The correspondence between the secondary structure and the sub-peaks in the fitted maps is as follows: the α-helix structure corresponds to wave numbers between 1649–1657 cm^−1^; the β-sheet structure is characterized by wave numbers ranging from 1610–1640 cm^−1^ and 1673–1677 cm^−1^; the β-turn structure is reflected by wave numbers between 1659–1674 cm^−1^ and 1681–1696 cm^−1^; the random coil structure is characterized by wave numbers within the range of 1641–1649 cm^−1^ [17,48]. According to the data depicted in Figure 1, the average α-helix content of the three soybean protein isolates was 15.54%. The range of variation was between 14.94% and 15.92%, with a coefficient of variation of 4.90%. It is worth noting that the highest α-helix content recorded was 970 W, and there were no significant differences observed among the three soybean protein isolates. The average β-sheet content was 40.93%, with a fluctuation range of 37.80–42.42%, and a coefficient of variation of 20.78%. The β-sheet content exhibited a mean value of 40.93%, with a range of variation between 37.80% and 42.42%. The coefficient of variation was calculated to be 20.78%. The β-sheet content reached its peak in the sample labelled as 950 E, whereas the sample labelled as 970 W demonstrated the highest β-turn content. In addition, the sample labelled as 950 E displayed the highest β-turn content, while the sample labelled as 970 W showed the highest β-turn content as well. The mean value of random coil content was 15.79%, the range of variation was 15.30~16.05%, the coefficient of variation was 2.16%, and the highest random coil content was 970 W. To summarize, there were no notable variations in the content of α-helix, β-sheet, β-turn, and random coil among the three soybean protein isolates. From this, it can be inferred that they are prepared by the same process (alkaline extraction and acid precipitation) for soybean protein isolates.

### 2.2. Texturized Vegetable Protein

#### 2.2.1. Textural Properties

Textural properties are the most important indicator of texturized vegetable protein, which can vary greatly with small changes in production operations, so it is necessary to determine their textural properties out of concern for the quality acceptability of the texturized vegetable protein [47,49,50]. Table 3 shows the results of the textural properties of the texturized vegetable proteins, which include hardness, adhesiveness, resilience, cohesion, springiness, gumminess, and chewiness. The mean hardness value of the low-moisture texturized vegetable proteins was 402.7 g, the coefficient of variation was 7.9%, and the maximum was 970 W. The mean value of adhesiveness was −1.4 g·s and the coefficient of variation was −53.7%. There was no significant difference between the means of the three texturized vegetable proteins. The mean value of resilience was 25.0%, the coefficient of variation was 3.4%, and the maximum was 971 W. The mean value of cohesion was 0.7, the coefficient of variation was 1.4%, the maximum was 971 W, and the minimum was 970 W. The mean value of springiness was 74.5% and the coefficient of variation of 3.8%, with the largest being 950 E and the smallest being 970 W. The mean value of gumminess was 289.7 and the coefficient of variation of 6.6%, with the largest being 970 W and the smallest being 950 E. The mean value of chewiness was 229.6, with a range of 208.6 to 249.8, with a coefficient of variation of 6.7%, and with a maximum for 970 W and a minimum for 950 E.

As a reference, texturized mung bean protein has a hardness of 18,690.53 g, springiness of 0.28, cohesion of 0.94, chewiness of 4087.56, and resilience of 0.65 [47]. In contrast, our texturized vegetable proteins have lower hardness, chewiness, and resilience, but more springiness and higher cohesion. This difference is partly due to the characteristics of the different raw materials, and partly to the way of rehydration (using different water temperatures and times). In general, high hardness and chewiness values will positively influence consumer acceptance of texturized vegetable proteins [13]. However, our texturized vegetable proteins are softer and more elastic and have a different texture and taste than real meat. It can be concluded that there are differences in the texturized vegetable proteins extruded from soybean protein isolates with different functional properties.

#### 2.2.2. Degree of Texturization

**Figure 2:**
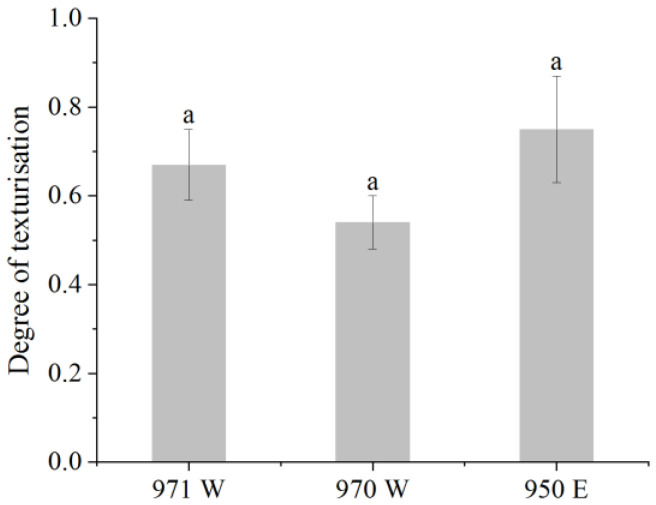
Degree of texturization of three texturized vegetable proteins. Different small letters above the bars indicate statistical differences (*p* < 0.05).

Determining the degree of texturization is a critical factor in evaluating meat analogues, as it indirectly influences consumer acceptance of texturized vegetable proteins [47]. The degree of texturization was measured by the ratio of transverse and longitudinal, indicating the size of the degree of texturization of texturized vegetable proteins, and the higher the value indicated, the better the degree of texturization. Figure 2 shows the results. The average value of the degree of texturization was 0.65, with a variation of 0.54 to 0.75, and the coefficient of variation was 29.45%, with no significant difference among three texturized vegetable proteins. The longitudinal cutting strength (F_l_) of the texturized vegetable proteins surpassed that of the transverse cutting strength (F_t_), indicating the development of fibrous structures. The researchers found that the shear ratio of transverse to longitudinal was less than 1 because the presence of a parallel fibrous network requires a higher force than the perpendicular network to cut the texturized vegetable proteins [51]. Compared to texturized mung bean protein (with a value of 1.23 for the degree of texturization) [47], we have a lower value for the degree of texturization, and according to the textural properties, the three texturized vegetable proteins have lower structural properties, and the textural properties are more consistent with the degree of texturization.

#### 2.2.3. Free SH and S-S Content

The free SH and S-S contents of texturized vegetable proteins are shown in Table 4. The degree of fibrillation in extruded texturized vegetable proteins during the extrusion cooking process is primarily influenced by the extent of protein cross-linking, which is facilitated by the formation of S-S through intramolecular and intermolecular sulphide exchange reactions [15]. As Table 4 shows, the average of total SH was 139.31 μmol/g, with a variation of 120.71~151.95 μmol/g and a coefficient of variation of 21.23%, and the mean value of free SH was 3.54 μmol/g, with a variation of 3.30~3.88 μmol/g and a coefficient of variation of 11.21%. The mean value of S-S was 67.89 μmol/g, with a variation of 58.42 to 74.32 μmol/g and a coefficient of variation of 21.79%. The total SH, free SH, and S-S contents were consistent for all three texturized vegetable proteins, showing no variability. This is because under the same process of extrusion, the degree of denaturation and cross-linking of proteins and the intramolecular and intermolecular interactions of proteins are the same in the barrel of the machine [52].

#### 2.2.4. Microstructure

**Figure 3:**
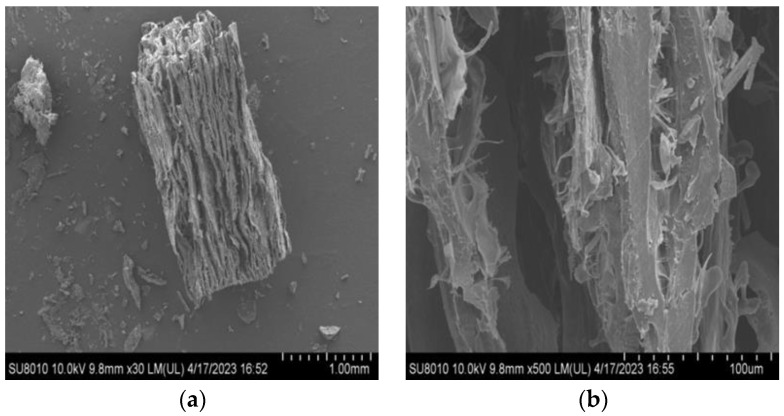
Longitudinal section of 950 E product (rehydrated); (**a**) represents 30×; (**b**) represents 500×.

**Figure 4:**
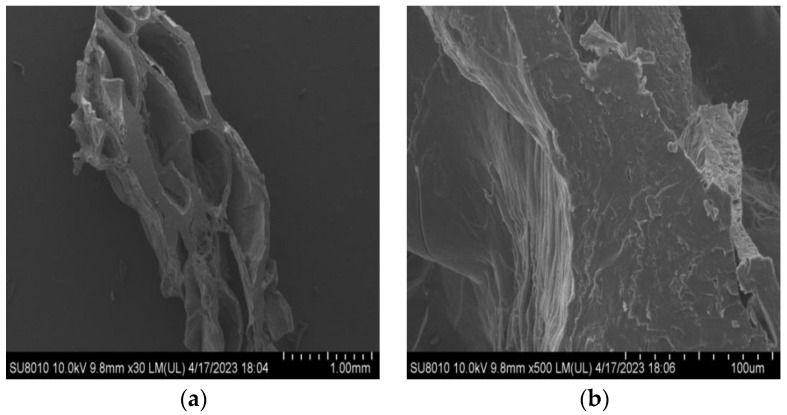
Longitudinal section of 950 E product (non-rehydrated); (**a**) represents 30×; (**b**) represents 500×.

SEM can visualize the microstructural changes in the sample, providing high-resolution images and information on protein aggregation after the extrusion process [53]. Using the 950 E texturized vegetable protein as an example, Figure 3 and Figure 4 depict the microstructure of the texturized vegetable proteins produced under extrusion cooking in rehydrated and non-rehydrated forms, respectively. Figure 3 shows that the rehydrated texturized vegetable proteins have pores of different sizes, the surface becomes irregular, and the tiny fibres are swollen and interconnected. The pore size of non-rehydrated texturized vegetable proteins is larger than that of rehydrated ones, and the fibres are relatively tightly arranged, as shown in Figure 4. In summary, at the barrel temperature (150 °C), the friction and shear forces between the screw and the barrel promote the degree of protein texturization and are conducive to fibre formation. The researchers also observed the formation of visible fibres when the barrel temperature was 149 °C [20]. The researchers found that the fibre structure of mung bean protein aligns with shear flow during extrusion, resulting in irregular protofibres that tightly connect to form a network-like structure in both hydrated and non-hydrated forms [47]. Comparison of the hydrated texturized vegetable protein (Figure 3) with the hydrated oat-pea meat analogue [13] shows that they both have a spongy appearance and shape, and both are classically low-moisture extruded texturized vegetable proteins. They both have a distinct laminar fibrous structure and are observed by electron microscopy at 500 times magnification as elongated fibres that also contain pores of various sizes. The present study is in agreement with literature reports.

### 2.3. Correlation of Soybean Protein Isolates with Texturized Vegetable Proteins

#### 2.3.1. Correlation of Functional Properties of Soybean Protein Isolates with Texturized Vegetable Proteins

The correlation between the functional properties of soybean protein isolates and texturized vegetable proteins can be seen (Table 5). The EAI of soybean protein isolates was significantly negatively correlated with hardness, highly significant positively correlated with adhesiveness and springiness, and significantly negatively correlated with gumminess and chewiness of texturized vegetable proteins. A high EAI value indicates that the soybean protein isolate has good emulsification and stability to form stable emulsions. This may be easier to chew and softer (i.e., lower hardness) in the preparation of texturized vegetable proteins. Soybean protein isolates with high EAI values may exhibit better adhesion and elasticity in the preparation of texturized vegetable proteins. Therefore, adhesiveness and springiness showed highly significant positive correlation with EAI. High EAI values of soybean protein isolates may lead to tighter binding of proteins in extruded texturized vegetable proteins and formation of stiffer network structures. With the action of heating and screw cutting, disulfide bonds are broken, which may reduce the gumminess and chewiness of the texturized vegetable proteins, so gumminess and chewiness showed a significant negative correlation with EAI. The ESI of soybean protein isolates showed significant positive correlation with springiness and degree of texturization of texturized vegetable proteins and significant negative correlation with gumminess. High ESI values indicate that soybean protein isolates dissolve well in water and form stable solutions. This may result in the preparation of texturized vegetable proteins with better springiness and degree of texturization; however, high ESI values may also result in texturized vegetable proteins that have more adhesiveness, reducing chewiness [17]. Therefore, the emulsification characteristics of soybean protein isolates have an effect on the textural properties of texturized vegetable proteins.

The FC of soybean protein isolates showed a significant positive correlation with the resilience of texturized vegetable proteins. The FC of soybean protein isolate is a measure of the solubility of proteins, and a high FC value indicates that soybean protein isolate has high solubility [54,55]. In the process of preparing texturized vegetable proteins, the high solubility of soybean protein isolate promotes the combination with other components (e.g., polysaccharides, fats, etc.) and the formation of a gel or network structure with good elasticity. This gel or network structure is able to rebound under stress, thus improving the elasticity and resilience of the texturized vegetable proteins. The FS showed significant positive correlation with springiness and degree of texturization of texturized vegetable proteins, highly significant negative correlation with gumminess, and significant negative correlation with chewiness. This is mainly related to the thermal denaturation of proteins [56,57]. When the barrel temperature reaches a set temperature, the raw material hydrates with water, and under the rotation of the twin screw, it will trigger the unfolding of the peptide chain and rearrangement to form protein aggregates inside the barrel [57]. When protein molecules come into contact with the air-water interface, they are attracted and adsorbed, causing the interfacial tension to decrease. The peptide chains within the protein interact with each other through intramolecular and intermolecular forces, resulting in the formation of a two-dimensional network [35]. This process facilitates the formation of enhanced foam stability. The FS is thus positively correlated with springiness and degree of texturization and negatively correlated with gumminess and chewiness.

#### 2.3.2. Correlation of Amino Acid Content with Texturized Vegetable Protein

As shown in Table 6, Thr showed a significant negative correlation with the adhesiveness and springiness of the texturized vegetable protein. When Thr is present in high levels, it may interact with other amino acids in proteins, leading to increased mutual attraction between protein molecules, and thus increased adhesion of texturized vegetable proteins. Met presents a significant negative correlation with the adhesiveness of the texturized vegetable protein. Met is a sulphur-containing amino acid that plays an important role in protein stability and conformation. Met can form sulphur bonds in proteins, and such bonds can stabilize the spatial structure of proteins. When the content of Met is high, the number of sulphur bonds in proteins increases, which may lead to an increase in the mutual attraction between protein molecules, thus decreasing the adhesiveness of the texturized vegetable proteins. Arg presented a significant negative correlation with the free SH of the texturized vegetable protein. Arg is an amino acid containing amino and carboxyl groups that can participate in the formation of hydrogen and ionic bonds, and an increase in its content enhances the stability and structural compactness of proteins. SH is a sulfhydryl group (sulfhydryl group) in proteins, which can form thiol bonds and also plays an important role in protein stability and conformation. Further studies are needed to determine the exact mechanism as to how the amino acids in the above proteins affect the quality of the texturized vegetable proteins.

Although the correlation between amino acid content and texturized vegetable proteins was not statistically significant, the researchers observed the effect of L-cysteine addition on the functional and structural properties of texturized pea proteins, which significantly affects the textural properties and fibrillarity of texturized pea proteins by altering the cross-linking of the disulfide bonds between pea protein molecules [43]. Similarly, L-cysteine was added to wheat flour for extrusion, which is consistent with studies of L-cysteine addition to pea protein isolate [58]. Previous studies have reported that hydrolysis of proteins occurs during extrusion processing, resulting in varying degrees of loss of various amino acids, with relatively high amino acid losses due to the short processing time and low moisture content [59]. Nevertheless, during extrusion cooking, moisture plays a protective role in minimizing amino acid losses. This is because the material has the capability to decrease shear stress and dissipate mechanical energy within the extruder [60].

#### 2.3.3. Correlation of Secondary Structure Content with Texturized Vegetable Protein

The degree of texturization is significantly negatively correlated with α-helix, as demonstrated in Table 7. Similarly, random coils have a significant negative correlation with resilience and cohesion. The α-helix represents the protein’s spatial structure and orderliness, and its stability is primarily sustained by hydrogen bonds [61]. Soybean protein isolates are processed through temperature, pressure, and shear forces in the extruder barrel. These forces lead to the destruction or weakening of hydrogen and disulfide bonds in the protein molecule. Consequently, the molecule partially unfolds, and the hydrogen bond breakage causes a gradual change in the protein’s spatial structure from ordered to disordered [42]. Therefore, rehydration of the product leads to weakened protein-water interaction forces.

## 3. Materials and Methods

### 3.1. Materials

We purchase three kinds of soybean protein isolates, 970 W, 971 W, 950 E, and soybean meal (Shandong Yuwang Group, Yucheng, China) and corn starch (Heilongjiang Pengcheng Biochemical Co., Ltd., Qiqihar, China). Protein content was determined by the Kjeldahl method with a conversion factor of 6.25. The protein content of soybean isolates from 971 W, 970 W, and 950 E were 82.84, 82.35, and 82.79 g/100 g.

### 3.2. Low-Moisture Extrusion Process

For the extrusion process, a Brabender DSE-25 pilot-scale food extruder with co-rotating (Brabender GmbH and Co., Duisburg, Germany) intermeshing twin screws was employed. The extruder has a screw diameter of 25 mm and a screw length to diameter ratio of 20:1. The ratio of raw materials used for extrusion was 6:3:1 for soybean protein isolate, soybean meal, and corn starch. The pump and feeder were calibrated and adjusted to feed moisture of 20%. On a dry basis, the yield was 1.8 kg/h. The temperatures in the extruder barrel remained constant at 60 °C, 110 °C, 130 °C, 140 °C, and 150 °C.

### 3.3. Functional Properties of Soybean Protein Isolates

#### 3.3.1. Water Holding Capacity (WHC)

With slight adjustments, this method was used [37]. Dissolve 0.1 g of protein sample (labelled M_1_) in phosphate buffer solution (0.01 mol/L, 5 mL). Weigh the centrifuge tube beforehand and record its mass (labelled as M_2_). Transfer to a 10 mL centrifuge tube, vortex for 3 min at room temperature, and centrifuge at 5000 rpm for 20 min. Remove the supernatant and take note of the mass (labelled as M_3_). The measurements were conducted in triplicate. The WHC of the raw material is calculated using the following formula:

$$WHC=\frac{M_{3}-M_{2}-M_{1}}{M_{1}}\times 100\%$$

#### 3.3.2. Emulsifying Properties

The Emulsification Activity Index (EAI) and the Emulsification Stability Index (ESI) are two important properties of proteins that are related to emulsification. This method was used for the determination of emulsification properties (EAI and ESI) with slight modifications [17]. In a centrifuge tube, soybean protein isolate (0.30 g) was combined with water (30 mL) and stirred for 10 min. Following this, soybean oil (10 mL) was added and homogenized at a speed of 10,000 rpm for 1 min. At 0 min, a sample (50 μL) was collected and added to a mixed 1% sodium dodecyl sulphate (SDS) solution (5 mL). After 10 min, the sample (50 μL) was collected and added to a mixed 1% SDS solution (5 mL). Sample absorbance at 500 nm was measured by UV spectrophotometer (UV-9000 Metash Shanghai Yuan Analytical Instrument Co., Shanghai, China). The following formulas were used to calculate EAI and ESI.

$$EAI(m^{2}/g)=\frac{T\times 2\times N\times A_{0}}{10000\times ∁\times \emptyset }$$

$$ESI(min)=\frac{A_{0}\times ∆T}{A_{0}-A_{10}}$$

The equation is as follows: T = 2.303; N represents the dilution factor; A_0_ denotes the initial absorbance before homogenization; C represents the concentration of soybean protein isolate (g/mL) prior to emulsion formation; ø represents the volume of the emulsion, which is equivalent to 1/4; A_10_ denotes the absorbance of the emulsion after 10 min of homogenization; ΔT is a time-varying value of 10 min.

#### 3.3.3. Foaming Properties

The foam capacity (FC) and foam stability (FS) were based on this method with minor modifications [17]. Weighing 0.30 g of soybean protein isolate, it was then combined with 30 mL of phosphate buffer solution (PBS) at pH 7.0 in a centrifuge tube. Continue to homogenize for an additional 1 min at a speed of 10,000 rpm. Next, swiftly transfer the mixture to a 50 mL graduated cylinder for quantification and reassess the foam volume after a complete duration of 25 min. The calculation of FC and FS was conducted using the following method:

$$FC=\frac{V_{1}}{V}\times 100\%$$

$$FS=\frac{V_{25}}{V_{1}}\times 100\%$$

where: V is the initial volume of the solution (mL); V_1_ is the volume of the foam after 1 min of homogenization (mL); V_25_ is the volume of the foam after 25 min of resting (mL).

### 3.4. Content of Amino Acids

Following this method [47], proposed soybean protein isolate (0.1 g), 6 mol/L HCl (10 mL), and phenol (2 drops) were combined in a reaction tube. For 15 min after filling the reaction tube with nitrogen, it was subsequently sealed and incubated at a temperature of 110 °C in an oven for a period of 24 h. After the reaction tube was cooled to room temperature, the contents were passed through a cellulose filter paper for filtration. The resulting filtrate was then mixed with deionized water until a final volume of 50 mL was achieved. To supplement the filtrate, 1 mL of sodium citrate buffer with a pH of 2.2 was added. Before performing amino acid analysis on an automated amino acid analyser (Hitachi L-8900, Tokyo, Japan), the solution was further filtered using a syringe filter with a pore size of 0.22 µm.

### 3.5. Secondary Structure of Proteins

Refer to this method [62] with slight modifications. The first step was to collect the air background and then scan the potassium bromide background and deduct the background for the determination of the sample. Before conducting infrared spectral analysis, the sample (1 mg) and KBr (100 mg) were accurately weighed and placed into an agate mortar. Then, they were ground under an infrared lamp. After grinding, the mixture was compressed in a tablet press at a pressure of 14 kg for approximately 1 min. The resulting uniform and transparent sheets were analysed using a Bruker Equinox 55 spectrometer (Bruker Instrument Company, Karlsruhe, Germany) for infrared spectral analysis. Fourier Transform Infrared Spectrometer (FTIR) (Germany TENSOR 27 (ATR), Bruker, Karlsruhe, Germany) operating conditions: the infrared spectra were recorded in the wave number range of 400–4000 cm^−1^, with a resolution of 4 cm^−1^, signal scanning cumulative 64 times, each sample was measured 3 times, and the average value was obtained. The infrared curves were processed with the OMNIC 8.0 software (Thermo Fisher Scientific, Waltham, MA, USA) for the correction of the baseline, the normalization, and the Fourier deconvolution. PeakFitv4.12 software (Systat Software Inc., San Jose, CA, USA) was used to analyse and calculate the percentage of sub-peak structures in the amide I region.

### 3.6. Texturized Vegetable Protein

#### 3.6.1. Textural Properties

Based on this approach [49], the pre-treatment of the samples (three texturized vegetable proteins (971 W, 970 W, and 950 E)) involved hydrating them at room temperature for 90 min. After being drained using a sieve for 30 min, it is cut with a knife to a length of 20 mm. During the texture analysis conducted in TPA mode, a cylindrical aluminium probe (P/36R, Ø 35 mm) installed in the Texture Analyser (TA.XT2, Stable Micro Systems, Godalming, UK) was utilized to compress the sample to 50% of its initial height. The test parameters for the plus texture analyser were configured as follows: the pre-test speed was set to 2 mm/s, the test speed was set to 1 mm/s, the post-test speed was set to 2 mm/s, the trigger force was set to 5 g, and the time interval between two compressions was set to 5 s. Each sample was replicated five times.

#### 3.6.2. Degree of Texturization

These methods were referenced with slight modifications [47]. The test method was as follows: the samples were trimmed to a length of 20 mm and placed on the sample stage for testing. Longitudinal (parallel to the direction of extrusion) and transverse (perpendicular to the direction of extrusion) shear determinations were made on the sample using the Texture Analyser’s (TA.XT2, Stable Micro Systems, Godalming, UK) A-CKB probe in shear mode. The physical property tester was operated in shear mode with the following parameters: 2 mm/s before the test, 1 mm/s during the test, and 2 mm/s after the test while maintaining 75% shear. The measurements were repeated five times for each sample, and the average value was calculated from the results. The degree of texturization was calculated using the following formula:

$$Degree\;of\;texturization=\frac{F_{t}}{F_{l}}$$

In the given formula, the symbol F_t_ represents the shear force in the transverse direction, while the symbol F_l_ represents the shear force in the longitudinal direction.

#### 3.6.3. Free Sulfhydryl and Disulfide Content of Proteins (Free SH and S-S Content)

Free SH were measured using the 5,5-dithiobis (2-nitrobenzoic acid) (DTNB) method as described by [63] and Ellman’s method [37], in which 4 mg of DTNB was added to 1 mL of Tris-Gly in solution at a pH of 8.0. In the experiments, the sample (3 mg) was dissolved in 5 mL of Tris-Gly-EDTA buffer solution and reacted for 30 min with shaking every 10 min. After 30 min, 40 μL of DTNB was added, shaken, and mixed, and the reaction was wrapped in tinfoil and reacted at 25 °C for 30 min. The reaction was centrifuged at 25 °C (13,600 *g* for 10 min), and the supernatant was analysed for absorbance at 412 nm. The reagent blank was subtracted from the sample. The Tris-Gly-EDTA buffer solution consisted of Tris (0.086 mol/L), Gly (0.09 mol/L), and EDTA (0.04 mol/L), and the pH was adjusted to 8.0 with hydrochloric acid. Free SH content was calculated as follows:

$$Free\;SH\;(\mu mol/g)=\frac{73.53\times A_{412}\times D}{C}$$

In the provided formula, the value of 73.53 is derived from the ratio 106/13,600. The variable A_412_ represents the absorbance measured at 412 nm. The variables C and D denote the protein concentration (mg/mL) and the dilution factor, respectively.

Determination of S-S content: Sample (3 mg) was added to 5 mL Tris-Gly-EDTA, then 0.05 mL of β-mercaptoethanol (β-ME), held at 25 °C for 1 h, then added 12% trichloroacetic acid (TCA) (10 mL) and continued to be held at 25 °C for 1 h, centrifuged (4000 rpm, 10 min), and the supernatant was poured off and the precipitate was retained. Dissolve the precipitate in 5 mL of 12% TCA, wash the precipitate, continue centrifugation, pour off the supernatant, and keep the precipitate. The precipitate was dissolved in 5 mL of Tris-Gly-EDTA, 40 μL of DTNB was added, and the reaction was carried out for 30 min to measure the absorbance at 412 nm. The content of S-S was calculated according to the following formula:

$$S\text{-}S\;(\mu mol/g)=\frac{73.53\times A_{412}\times \frac{D}{C}-SH}{2}$$

where 73.53 = 106/13,600, D denotes dilution factor, and C denotes protein concentration (g/L).

#### 3.6.4. Microstructure

We used a scanning electron microscope (SEM) (Hitachi SU8010, Tokyo, Japan) to analyse the microstructure, following the procedure outlined with minor adjustments [47]. Prior to observation, both hydrated and non-hydrated samples were subjected to freeze-drying and further coated with a layer of gold. The observations were carried out at 15 kV accelerating voltage, using a magnification of 30× and 500× for all images.

### 3.7. Data Analysis

Results are expressed as the mean ± standard deviation. The statistical software SPSS 24.0 (IBM, Armonk, NY, USA) was utilized to conduct both one-way ANOVA (analysis of variance) and Pearson correlation analysis. Graphs were created using Origin 2010 software (OriginLab, Northampton, MA, USA). The mean values of the samples showed significant differences at a significance level of *p* < 0.05.

## 4. Conclusions

In this study, three different texturized vegetable proteins were prepared by low-moisture extrusion using three different soybean protein isolates as raw materials in order to investigate the relationship between the soybean protein isolate and textural properties of texturized vegetable protein. The results showed that soybean protein isolates with different functional properties (e.g., WHC, foaming properties, emulsifying properties) had different effects on the textural properties of the texturized vegetable proteins. However, the functional properties of proteins had more significant effects on the textural properties of texturized vegetable proteins than the primary (amino acid) and secondary structures of proteins. Observation by scanning electron microscopy (SEM) revealed that the texturized vegetable protein fibres were uniformly distributed in one layer and had pores of different sizes. These results not only confirmed that texturized vegetable proteins have desirable physical properties and fibre structure, but also indicated that texturized vegetable proteins have great potential as an excellent choice and provide a theoretical basis for the processing of texturized vegetable proteins as raw materials.

## Figures and Tables

**Table 1:** Functional properties of soybean protein isolates.

Soybean Protein Isolates	WHC (%)	Emulsifying Properties		Foaming Properties	
		EAI (m^2^/g)	ESI (min)	FC (%)	FS (%)
970 W	6.37 ± 0.31 b	19.62 ± 0.55 c	16.30 ± 0.29 a	1.40 ± 0.02 b	0.28 ± 0.01 a
971 W	6.83 ± 0.15 a	22.19 ± 0.64 b	19.08 ± 2.96 a	1.68 ± 0.02 a	0.31 ± 0.03 a
950 E	6.33 ± 0.21 b	25.28 ± 0.03 a	20.35 ± 1.65 a	1.34 ± 0.05 b	0.35 ± 0.06 a

Different letters following the data in the same column mean significant differences (*p* < 0.05).

**Table 2:** Amino acid content in soybean protein isolates.

Amino Acids (g/100 g)	970 W	950 E	971 W
Aspartic (Asp)	9.4 ± 0.6 a	9.0 ± 0.0 a	9.4 ± 0.6 a
Threonine (Thr)	3.1 ± 0.2 a	2.9 ± 0.0 a	3.0 ± 0.2 a
Serine (Ser)	4.3 ± 0.3 a	4.1 ± 0.0 a	4.2 ± 0.3 a
Glutamine (Glu)	16.0 ± 1.0 a	15.6 ± 0.0 a	15.9 ± 0.9 a
Proline (Pro)	3.9 ± 0.1 a	3.9 ± 0.00 a	3.9 ± 0.0 a
Glycine (Gly)	3.4 ± 0.2 a	3.1 ± 0.0 a	3.3 ± 0.2 a
Alanine (Ala)	3.4 ± 0.2 a	3.2 ± 0.0 a	3.4 ± 0.2 a
Valine (Val)	3.8 ± 0.2 a	3.6 ± 0.0 a	3.8 ± 0.2 a
Methionine (Met)	1.0 ± 0.0 a	0.9 ± 0.0a	1.0 ± 0.1 a
Isoleucine (Ile)	3.8 ± 0.2 a	3.6 ± 0.0 a	3.8 ± 0.2 a
Leucine (Leu)	6.3 ± 0.4 a	6.1 ± 0.0 a	6.3 ± 0.4 a
Tyrosine (Tyr)	3.0 ± 0.2 a	2.9 ± 0.0 a	3.0 ± 0.2 a
Phenylalanine (Phe)	4.2 ± 0.3 a	4.1 ± 0.0 a	4.2 ± 0.3 a
Lysine (Lys)	5.1 ± 0.3 a	4.8 ± 0.0 a	5.1 ± 0.3 a
Histidine (His)	2.1 ± 0.1 a	1.9 ± 0.0 a	2.1 ± 0.1 a
Argnine (Arg)	6.7 ± 0.2 a	6.0 ± 0.0 b	6.9 ± 0.1 a

Different letters following the data in the same row indicate significant differences (*p* < 0.05).

**Table 3:** Textural properties of three texturized vegetable proteins.

Textural Properties	970 W	950 E	971 W
Hardness (g)	457.7 ± 44.3 a	351.5 ± 16.5 b	398.8 ± 37.1 b
Adhesiveness (g·s)	−1.9 ± 0.7 a	−1.1 ± 0.7 a	−1.2 ± 0.7 a
Resilience (%)	24.1 ± 0.6 b	24.9 ± 1.2 ba	26.0 ± 0.8 a
Cohesion	0.7 ± 0.0 c	0.7 ± 0.0 b	0.7 ± 0.0 a
Springiness (%)	69.9 ± 3.9 c	79.2 ± 2.7 a	74.4 ± 1.9 b
Gumminess	320.8 ± 20.9 a	255.3 ± 12.6 c	293.1 ± 24.2 b
Chewiness	249.8 ± 24.7 a	208.6 ± 4.9 b	230.5 ± 18.3 ba

Different letters following the data in the same row indicate significant differences (*p* < 0.05).

**Table 4:** Free SH and S-S content.

Texturized Vegetable Proteins	Total SH (μmol/g)	Free SH (μmol/g)	S-S (μmol/g)
971 W	151.95 ± 26.24 a	3.30 ± 0.33 a	74.32 ± 13.26 a
970 W	145.27 ± 34.15 a	3.44 ± 0.58 a	70.92 ± 16.79 a
950 E	120.71 ± 27.66 a	3.88 ± 0.27 a	58.42 ± 13.93 a

Different letters following the data in the same column mean significant differences (*p* < 0.05).

**Table 5:** Correlation of functional properties of soybean protein isolates with texturized vegetable proteins.

	Hardness	Adhesiveness	Resilience	Cohesion	Springiness	Gumminess	Chewiness	Degree of Texturization	Total SH	Free SH	S-S
WHC	0.151	0.109	0.403	0.546	0.000	0.025	0.050	0.109	0.429	0.118	0.429
EAI	−0.783 *	0.900 **	0.150	0.450	0.817 **	−0.833 **	−0.817 **	0.633	−0.050	0.450	−0.050
ESI	−0.350	0.617	0.533	0.567	0.667 *	−0.683 *	−0.567	0.733 *	−0.033	−0.017	−0.033
FC	0.603	−0.067	0.703 *	0.577	−0.318	0.368	0.460	−0.117	0.377	−0.410	0.377
FS	−0.492	0.475	0.085	0.458	0.763 *	−0.814 **	−0.797 *	0.729 *	−0.458	0.153	−0.458

Note: Levels of significance *: *, *p* < 0.05, significant; **, *p* < 0.01, highly significant.

**Table 6:** Correlation between the content of amino acids in soybean protein isolates and texturized vegetable proteins.

	Hardness	Adhesiveness	Resilience	Cohesion	Springiness	Gumminess	Chewiness	Degree of Texturization	Total SH	Free SH	S-S
Asp	−0.300	0.033	−0.050	0.033	0.133	−0.267	−0.317	0.183	−0.250	−0.233	−0.250
Thr	0.444	−0.728 *	−0.293	−0.536	−0.703 *	0.653	0.619	−0.628	0.050	−0.360	0.050
Ser	−0.250	−0.083	−0.200	−0.200	0.017	−0.133	−0.183	0.033	−0.200	−0.183	−0.200
Glu	−0.300	0.033	−0.050	0.033	0.133	−0.267	−0.317	0.183	−0.250	−0.233	−0.250
Pro	−0.417	0.050	−0.417	−0.350	0.133	−0.233	−0.317	0.050	−0.283	−0.067	−0.283
Gly	−0.100	−0.283	−0.267	−0.317	−0.183	0.083	0.033	−0.200	−0.233	−0.133	−0.233
Ala	0.000	−0.500	−0.167	−0.350	−0.300	0.200	0.133	−0.183	−0.217	−0.117	−0.217
Val	−0.267	−0.133	−0.017	−0.033	0.017	−0.150	−0.217	0.100	−0.317	−0.133	−0.317
Met	0.550	−0.750 *	−0.133	−0.483	−0.567	0.550	0.583	−0.383	0.050	−0.400	0.050
Ile	−0.283	0.033	0.100	0.150	0.233	−0.367	−0.400	0.367	−0.350	−0.283	−0.350
Leu	−0.283	0.033	0.100	0.150	0.233	−0.367	−0.400	0.367	−0.350	−0.283	−0.350
Tyr	−0.217	−0.250	−0.167	−0.267	−0.100	−0.017	−0.083	−0.050	−0.267	−0.083	−0.267
Phe	−0.267	0.083	0.033	0.167	0.217	−0.350	−0.383	0.300	−0.283	−0.317	−0.283
Lys	−0.200	−0.033	0.150	0.233	0.183	−0.317	−0.350	0.333	−0.383	−0.300	−0.383
His	0.000	−0.500	−0.167	−0.350	−0.300	0.200	0.133	−0.183	−0.217	−0.117	−0.217
Arg	0.583	−0.600	0.283	0.167	−0.633	0.550	0.517	−0.350	0.067	−0.667 *	0.067

Note: Levels of significance *: *, *p* < 0.05, significant.

**Table 7:** Correlation between the secondary structure content of soybean protein isolates and texturized vegetable proteins.

	Hardness	Adhesiveness	Resilience	Cohesion	Springiness	Gumminess	Chewiness	Degree of Texturization	Total SH	Free SH	S-S
α-helix	−0.117	−0.017	−0.583	−0.467	−0.317	0.333	0.283	−0.733 *	0.017	0.350	0.017
β-sheet	0.117	−0.133	0.600	0.233	0.183	−0.217	−0.133	0.583	−0.133	−0.367	−0.133
β-turn	−0.133	0.217	−0.500	−0.117	−0.117	0.150	0.083	−0.533	0.033	0.333	0.033
random coil	−0.310	−0.276	−0.762 *	−0.745 *	−0.276	0.192	0.025	−0.418	−0.092	0.134	−0.092

Note: Levels of significance *: *, *p* < 0.05, significant.

## Data Availability

The raw data are contained within Supplementary Materials. The data presented in this study are available upon request from the corresponding author.

## Supplementary Materials

The following supporting information can be downloaded at: https://www.mdpi.com/article/10.3390/molecules28227465/s1, raw data.
