# Supplementary material for: Relationship between Soybean Protein Isolate and Textural Properties of Texturized Vegetable Protein
Source: Molecules. 2023 Nov 7;28(22):7465. doi: 10.3390/molecules28227465 (PMC10672934; doi:10.3390/molecules28227465)
Supplement: Supplementary file 1 [file molecules-28-07465-s001.zip › scanning electron microscope image.pptx]

## Slide 1
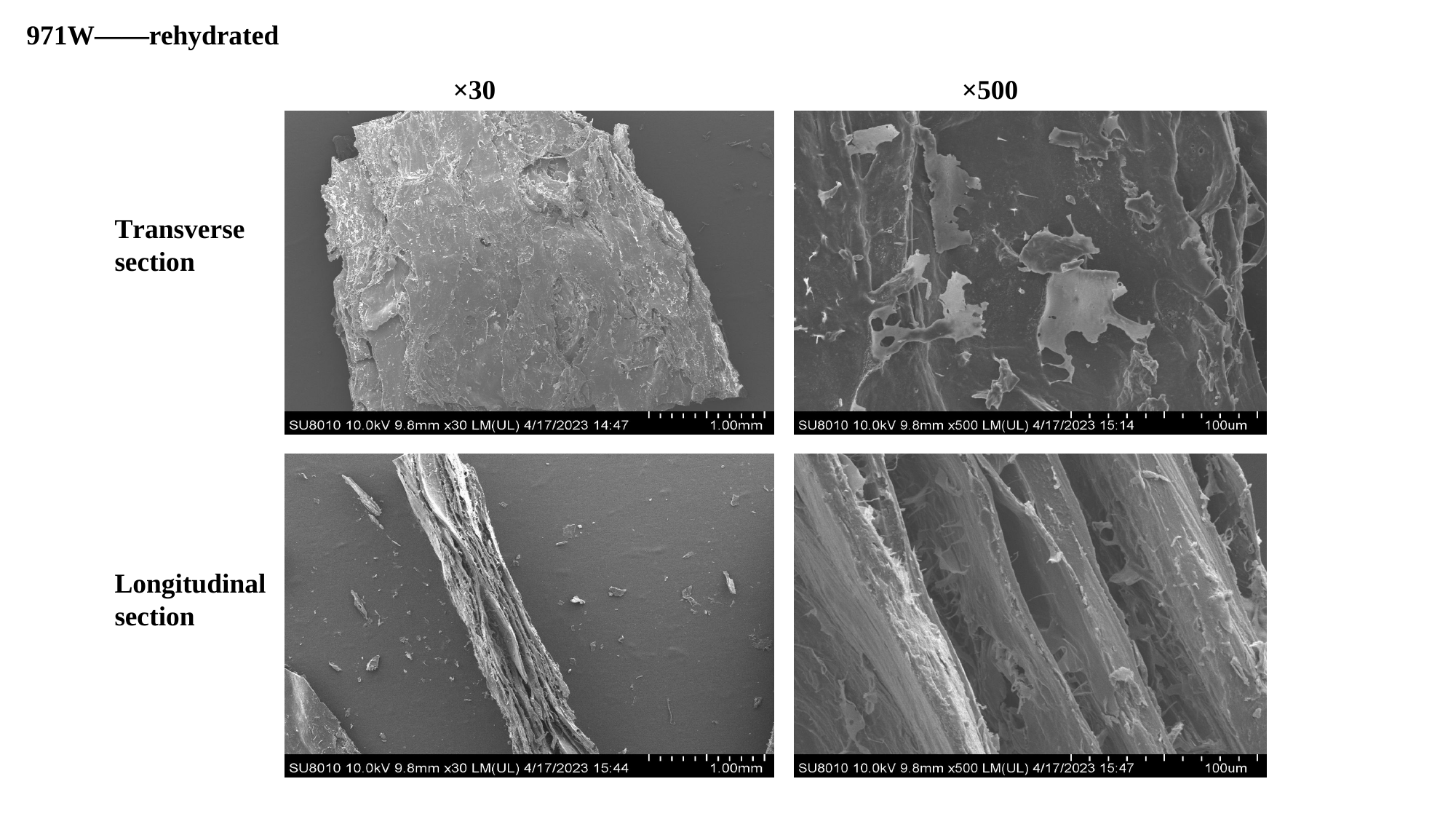

971W——rehydrated
×30
×500
Transverse section
Longitudinal section

## Slide 2
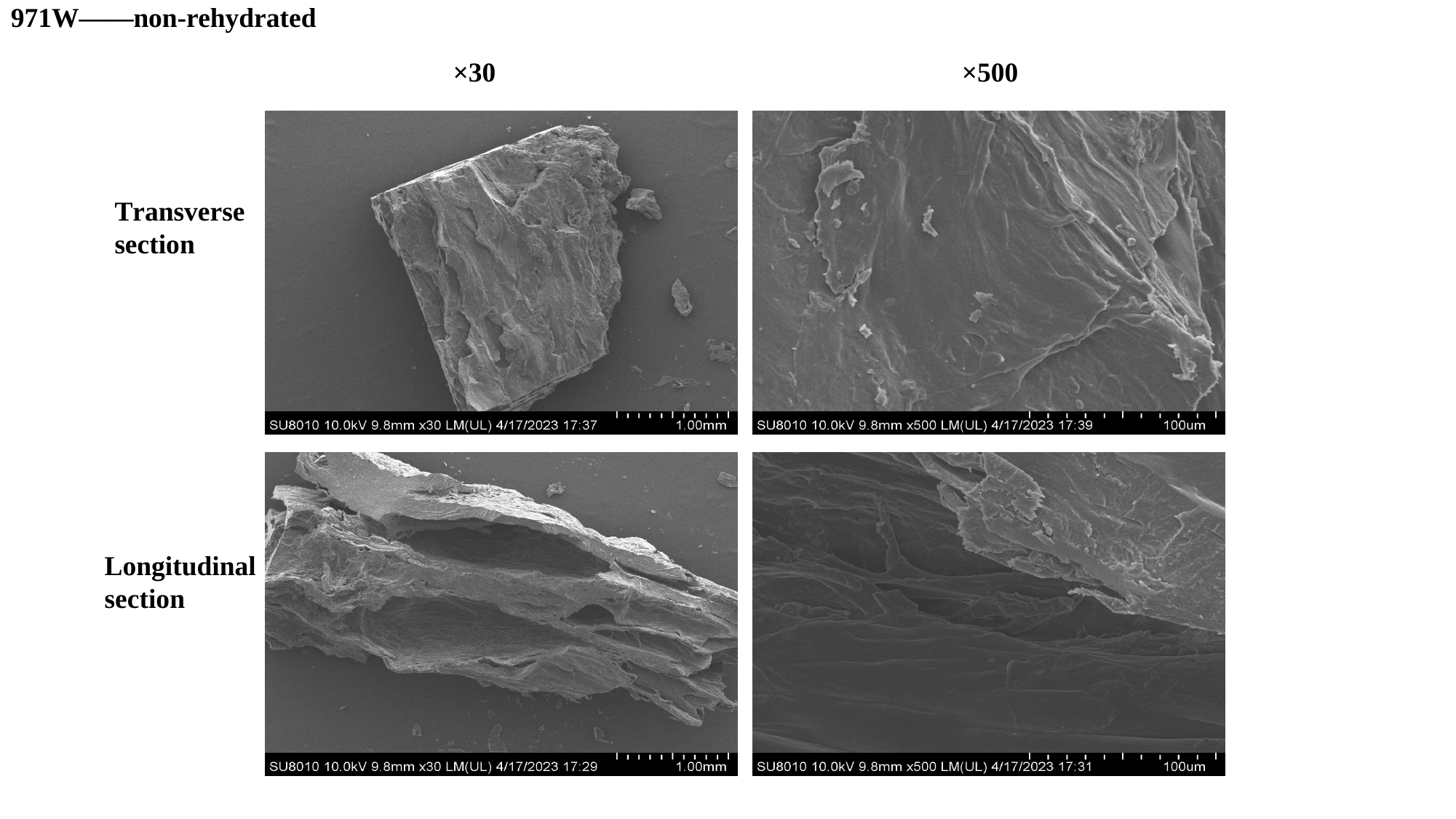

971W——non-rehydrated
×30
×500
Transverse section
Longitudinal section

## Slide 3
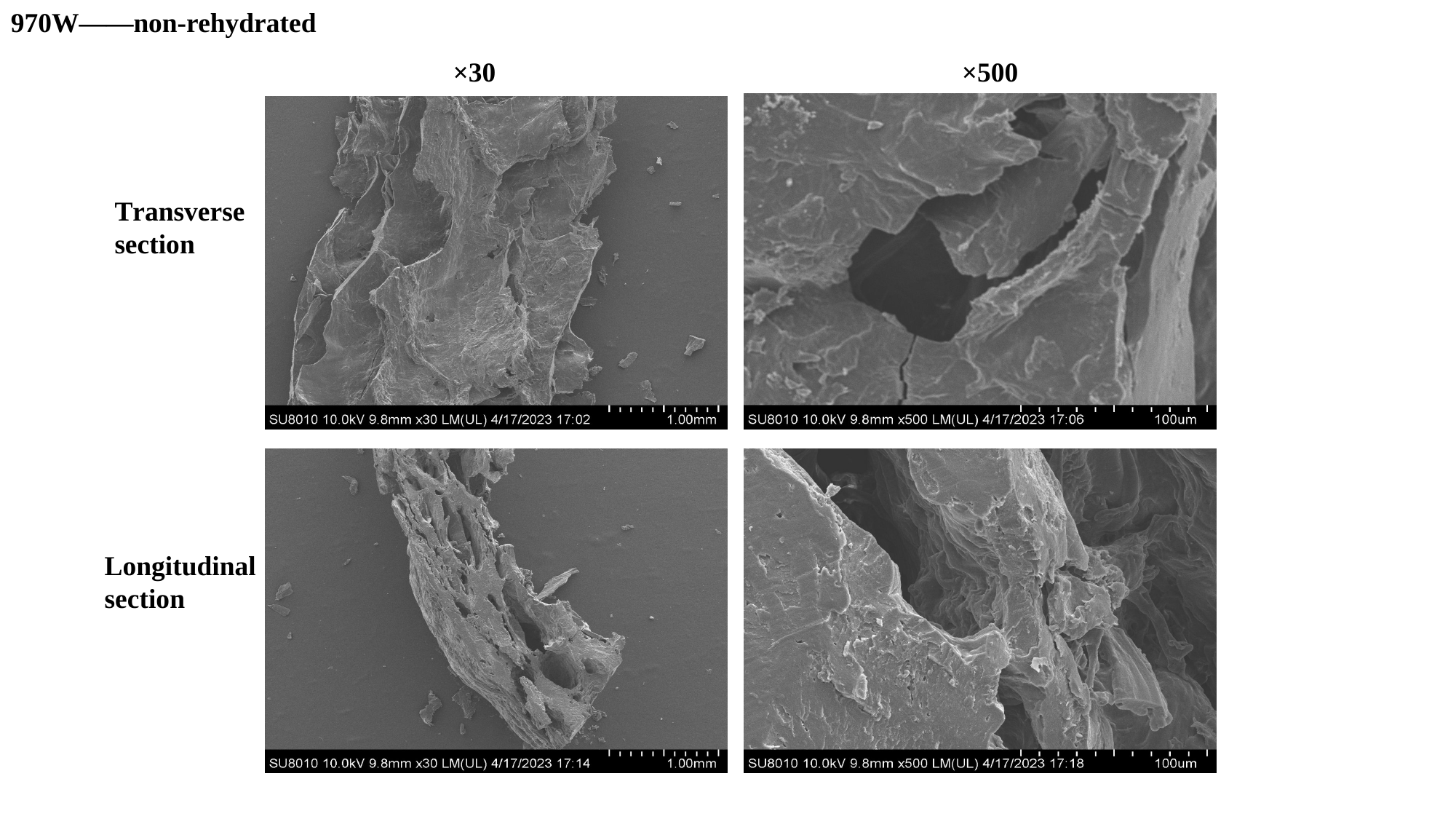

970W——non-rehydrated
×30
×500
Transverse section
Longitudinal section

## Slide 4
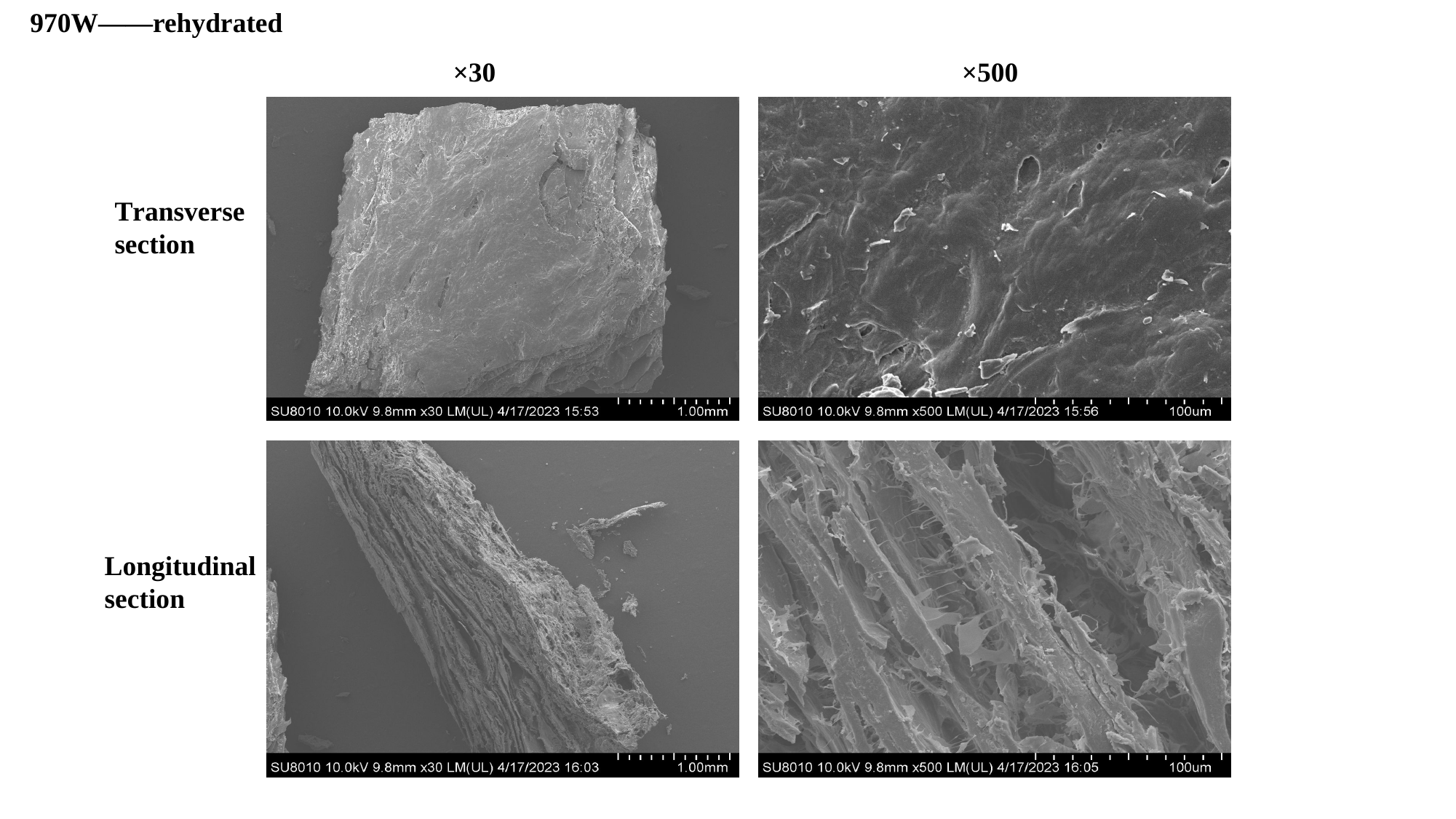

970W——rehydrated
×30
×500
Transverse section
Longitudinal section

## Slide 5
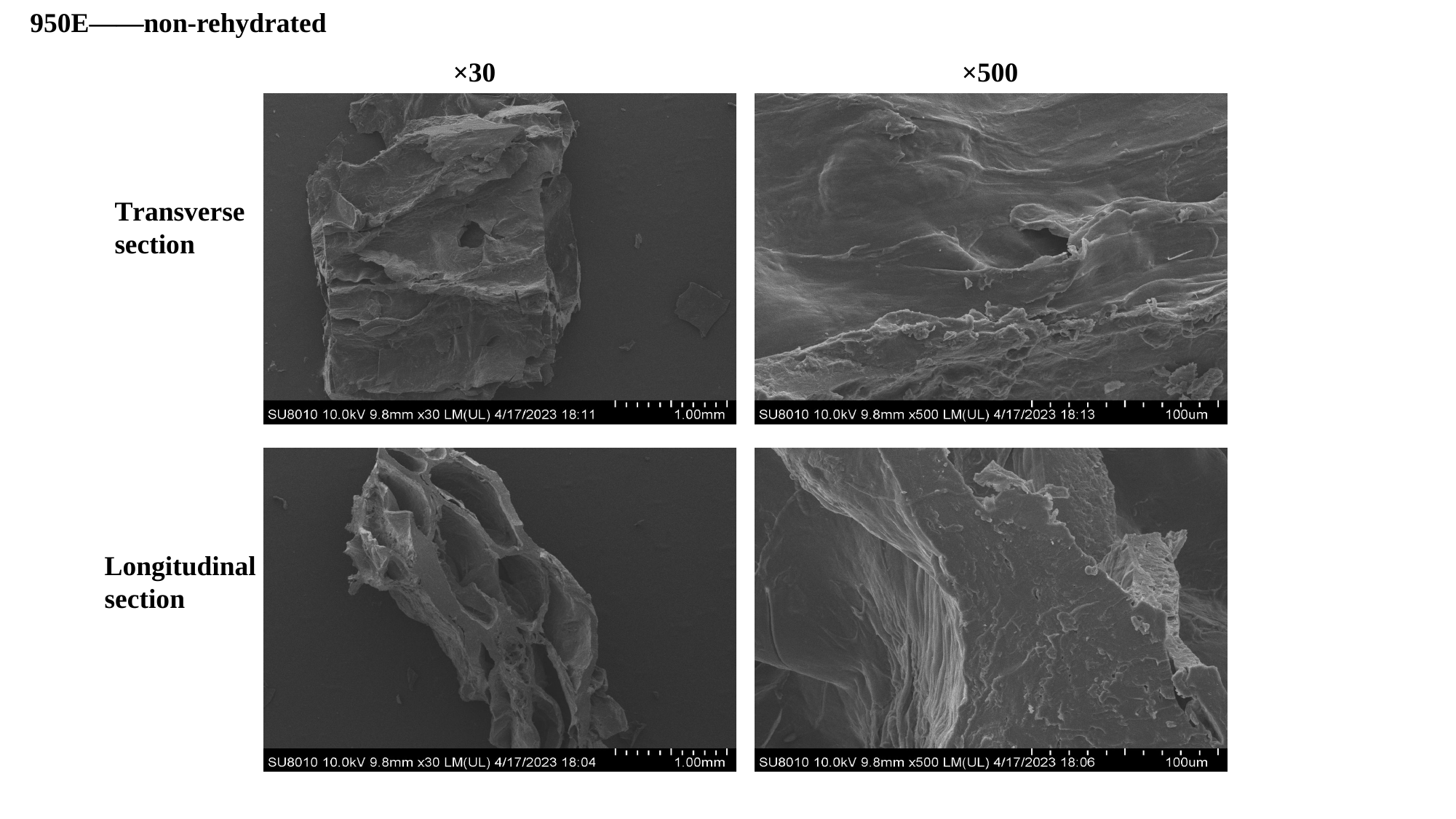

950E——non-rehydrated
×30
×500
Transverse section
Longitudinal section

## Slide 6
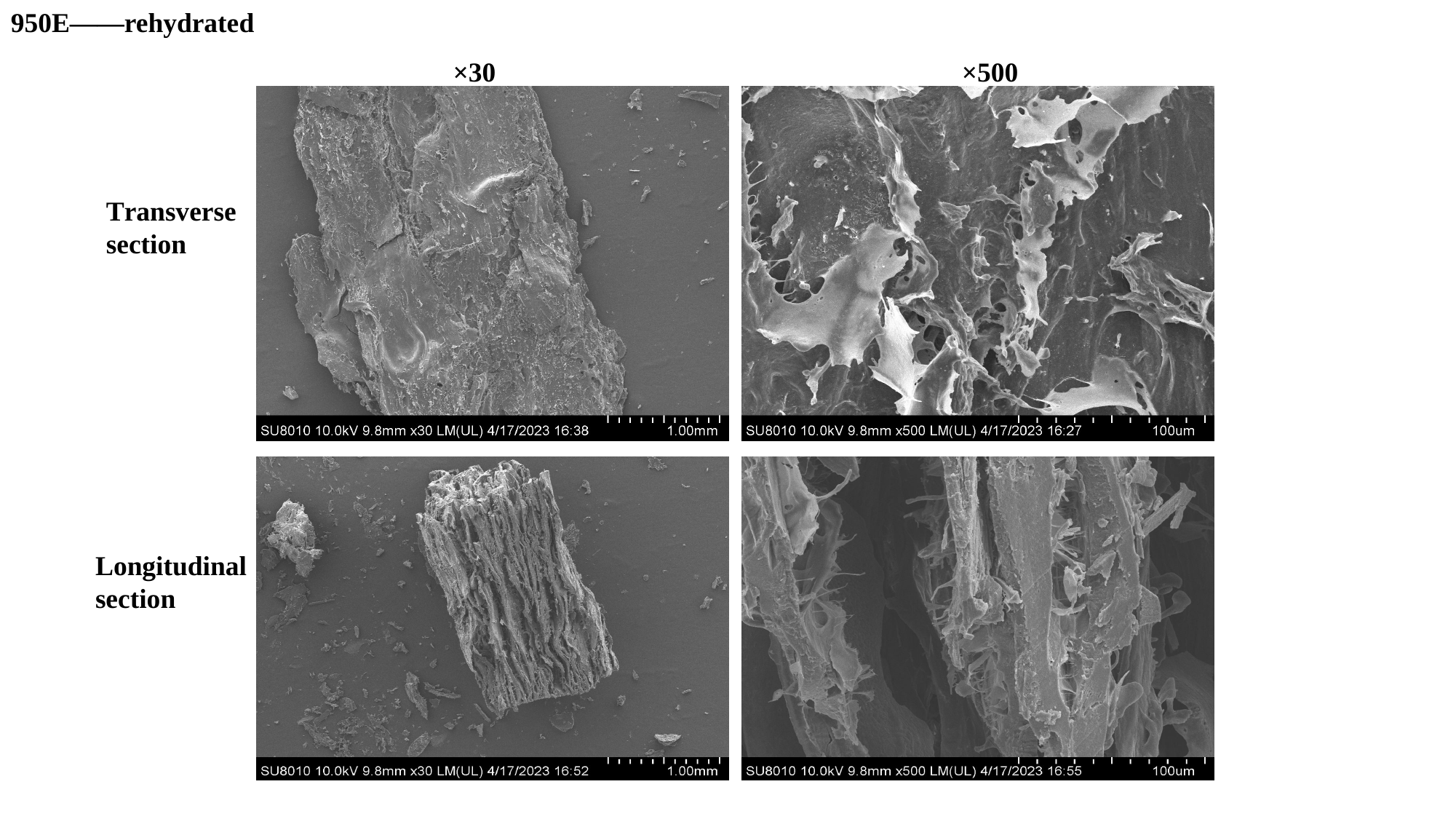

950E——rehydrated
×30
×500
Transverse section
Longitudinal section
